# The impact of adolescent achievement goal orientation on learning anxiety: The mediation effect of peer interaction

**DOI:** 10.3389/fpsyg.2023.1095498

**Published:** 2023-03-28

**Authors:** Haiyan Kong, Guangfeng Wang, Dazhi Cheng, Tian Li

**Affiliations:** ^1^School of Education, Shandong Women’s University, Jinan, China; ^2^School of Psychology, Qufu Normal University, Qufu, China; ^3^School of Psychology, Capital Normal University, Beijing, China; ^4^State Key Laboratory of Cognitive Neuroscience and Learning, IDG/McGovern Institute for Brain Research, Beijing Normal University, Beijing, China

**Keywords:** achievement goals, learning anxiety, peer interaction, social anxiety, adolescents

## Abstract

Learning anxiety is one of the most critical emotional disturbances, which also has a high incidence rate in adolescents. Peer interaction is critical and unique for adolescents. Although previous studies have found that achievement goal orientation has an important role in the development of learning anxiety, its mechanism has not been clarified. This study surveyed 470 adolescents (191 middle school students and 279 high school students; 211 boys) and established a structural equation model to explore the mediating role of peer interaction in the influence of achievement goal orientation on learning anxiety. Results showed that (1) there were significant gender differences in mastery-avoidance goal orientation, peer interaction, and learning anxiety, and there were grade differences in performance-approach goal and performance-avoidance goal orientations; (2) mastery-approach, mastery-avoidance, and performance-avoidance goal orientations directly predicted learning anxiety; and (3) social anxiety in peer interactions had a mediating effect on the influence of mastery-approach, mastery-avoidance, and performance-avoidance goal orientations on learning anxiety. The findings extend theoretical considerations by teasing out the process of peer interaction affecting the relationship between achievement goal orientation and learning anxiety. Additionally, the results have practical implications for the effective use of peer interaction to reduce learning anxiety.

## 1. Introduction

In recent years, the most difficult problem for students’ growth is psychological learning problems, and learning anxiety is one of the most critical emotional disturbances directly related to academic learning and achievement, which also has a high incidence rate in adolescents (Pekrun et al., 2009; Schnell et al., 2015; Chin et al., 2017). Learning anxiety is the most common negative emotional response of students in the learning process, which generally includes worry about negative evaluation by parents or teachers, anxiety and worry about exams, anxiety asking questions in class, avoidance and withdrawal tendencies, and symptoms evoked by the autonomic nervous system, such as insomnia and nightmares (Qiao et al., 1997). Learning anxiety is an emotional expectation of specific learning outcomes (such as outcomes that may threaten realistic or expected self-esteem and values; Wang and Downing, 2000) and a specific state of tension in the student population. There is evidence supporting the notion that the level of learning anxiety is higher in girls than in boys and that learning anxiety becomes more serious as people age. Learning anxiety not only affects adolescents academically but also leads to a poor mental state, low self-esteem, and other psychological problems. Therefore, it is of great practical significance to pay attention to the learning anxiety of adolescents and explore the mechanism influencing learning anxiety.

What exactly causes learning anxiety? Motivational psychologists and cognitive psychologists have been actively seeking to explain psychological and behavioral causes of learning anxiety, and among the potentially relevant proximal antecedents of achievement emotions (e.g., learning anxiety), students’ achievement goals are likely of pivotal importance (Pekrun et al., 2006; Huang, 2011; Anderman and Patrick, 2012; Linnenbrink-Garcia and Barger, 2014). Although research has focused on the influence of achievement goal orientation on learning anxiety, most of these studies have only examined the direct relationship between the two variables, and few empirical studies have explored the mechanism of action, especially the mechanism of school context.

The main task of secondary school students is to study; school is their main place of activity and peer relationships are important interpersonal relationships. Adolescents are particularly susceptible to peer influence. Adhering to social distancing may be particularly challenging for adolescents for whom interaction with peers is especially important (Andrews et al., 2020). In early adolescence, individuals gradually shift their emotional focus to close peers, spend more time with peers, and are more influenced by peers (Rudolph, 2008). As such, peers provide an important developmental context for their academic achievement (Rodkin and Ryan, 2012). So, does peer interaction, which is very important and unique for adolescents, affect learning anxiety? How exactly do they influence each other? This is the question this article explores. Therefore, the present study explored the influence of achievement goal orientation on learning anxiety and the mediating mechanism of peer relationships to clarify the mechanism of “how” achievement goal orientation affects adolescent learning anxiety. The results would have implications for interventions aimed at preventing and alleviating adolescent learning anxiety.

Based on previous empirical research, predictors of learning anxiety include learning motivation, peer comparison, considering the consequences of failure, low self-confidence, excessive worry about evaluation, parental sadness, lack of psychological preparation, and loss of self-worth (see Cassady and Johnson, 2002). Achievement goal orientation is an important component of learning motivation and is a competency-related goal to strive for in an achievement environment (Elliot, 2005). Achievement goal orientation is an individual’s perceived structure for achieving success, which is an important component of learning motivation. Achievement goal orientation develops with age but without gender differences (Guan et al., 2023); however, there are inconsistent findings (Meece et al., 2006b; Duchesne et al., 2014). Elliot and McGregor (2001) proposed a widely accepted 2 × 2 achievement goal orientation theory. In the theory, achievement goal orientation is divided into four categories: mastery-approach (focused on task-based or intrapersonal competence, e.g., “I want to learn as much as possible”), mastery-avoidance (focused on task-based or intrapersonal incompetence, e.g., “I may not learn all that there is to learn”), performance-approach (focused on normative competence, e.g., “I need to do better than others”), and performance-avoidance (focused on normative incompetence, e.g., “I need to avoid performing poorly”). Researchers argue that achievement motivation is an important motivation (Ames, 1992; Elliot, 2005) that can predict and regulate learning behavior, and it is an important factor in explaining students’ academic engagement and persistence (Anderman and Patrick, 2012). Achievement goal orientation is closely related to academic performance (Claver et al., 2020), academic mood, learning strategies, internal motivation, and learning interest (Hulleman et al., 2010). Previous studies found that the four categories of achievement goal orientation related differently to academic performance and learning emotions (Hulleman et al., 2010; Ranellucci et al., 2015; Goetz et al., 2016; Alhadabi and Karpinski, 2020). Specifically, mastery-approach positively predicted pride and enjoyment of learning and negatively predicted boredom and anger; performance-approach positively predicted anxiety, hopelessness, and shame (Pekrun et al., 2006); performance-avoidance positively predicted learning anxiety (Duchesne and Ratelle, 2010; Putwain and Symes, 2012). However, there is inconsistent evidence of weaker associations with performance-approach goals compared to mastery-approach goals (Mattern, 2005), which can be non-significant as shown in some studies (Cooper, 2014; Dull et al., 2015).

Although previous research indicates that achievement goal orientation may be beneficial in reducing children’s and adolescents’ learning anxiety, we do not know how achievement goal orientation affects learning anxiety. In particular, the mechanism by which school situational factors play a role in this relationship is not taken into account. Some researchers have pointed out that there are other mechanisms that have an effect on the association between achievement goal orientation and adolescents’ learning anxiety (e.g., Hall et al., 2016). Both constructivist theories and empirical research inspired by these researchers place peer interaction at the heart of many developmental processes (Tudge and Rogoff, 1999; Tenenbaum et al., 2019). According to the ecological systems theory (Bronfenbrenner, 1977), the behavior of peers in one environment will significantly affect the development of adolescents in another environment, and poor peer interaction may change the forms of activity of adolescents, thereby influencing the learning and development of adolescents (Berghout Austin and Draper, 1984; Bellmorea, 2011). For example, peer interaction can facilitate cognitive development (Topping et al., 2017) and achievement ability because students who seem to be capable are usually considered amiable rather than isolated (Berghout Austin and Draper, 1984). Therefore, some researchers have investigated the impact of peer interaction on this relationship.

Peer interaction refers to the transmission of information and emotions between individuals of the same age or individuals with similar levels of psychological development, such as individuals discussing or working on a task collaboratively. Critical features of peer interaction include the level of elaboration of help given and received and the appropriateness of responses to requests for help (Webb, 1989). Developmental research has indicated that peer interaction plays an important role in children’s socioemotional functioning, adjustment (Rubin et al., 2006; Chen, 2012), and adolescents’ learning (Webb, 1989; Rodkin and Ryan, 2012). First, good peer interaction reduces learning anxiety (Griffin and Griffin, 1998; Morgan, 2020), and social anxiety may increase adolescent developmental risk (Greca and Harrison, 2005). In studies on Chinese middle school students, interpersonal relationships, avoidance, and self-blame in the face of stressful events all positively predicted anxiety (Feng and Zhou, 2002), and interpersonal communication in real-life scenarios had an impact on students’ level of anxiety (Wang and Ding, 2003). Furthermore, peer support is associated with achievement goal orientation (Harter, 1996); however, previous studies have not reached a consistent conclusion. Some studies found stressful interpersonal relationships and stressful events were related to learning anxiety (Griffin and Griffin, 1998; Wang and Ding, 2003; Greca and Harrison, 2005). Mastery- and performance-approach goals were both positive predictors of deep learning, and performance-avoidance goals were negative predictors of peer relationships (Kuroda and Sakurai, 2011; Shim and Ryan, 2012; Liem, 2016). Levy et al. (2004) found that performance-avoidance-oriented students were inclined to show more maladaptive social outcomes, such as an unwillingness to cooperate with out-group members (Levy et al., 2004). However, satisfaction with peer interaction could significantly predict mastery-approach and mastery-avoidance goal orientations (Senko and Harackiewicz, 2005; Ciani et al., 2011; Hwang et al., 2017). Anderman and Anderman (1999) maintained that students who are concerned about peer interaction are more likely to be concerned about peer evaluations of their academic abilities; therefore, peer support could predict achievement goal orientation. Korpershoek et al. (2020) showed that students who “feel personally accepted, respected, included, and supported by others in the school social environment” are more likely to perform better in school (e.g., academic achievement) and show more favorable motivational (e.g., mastery goal orientations), socioemotional (e.g., self-concept and self-efficacy), and behavioral outcomes (e.g., behavioral, cognitive, and agentic engagement). Particularly, the female advantage in school might be primarily attributed to relational girls; they might experience more positive relationships with their peers, which could protect them against the decline in motivation and engagement in secondary schools (Burns et al., 2019). However, adolescents’ perceived peer support was not found to be significantly related to mastery goals or performance goals orientations (Wentzel, 1998).

In sum, previous studies have explored the relationship between achievement goal orientation and adolescent learning anxiety and peer interaction and adolescent learning anxiety, but no research has explored the relationship and mechanism among these three constructs. Furthermore, in studies investigating these relationships, there are no clear conclusions that can be made. In general, achievement goal orientations have been found to have no consistent correlation with learning anxiety. Moreover, the conflicting results and the correlation between the two variables lead us to wonder if other important variables are mediating the relationship between achievement goal orientation and learning anxiety. This question is related to the mechanism of action of learning anxiety in adolescents, which thus far has not been well studied. Notably, performance-avoidance orientation can increase peer interaction anxiety (Pintrich, 2000; Liem et al., 2008), but it is unclear whether peer interaction anxiety induces learning anxiety. Therefore, the research question is whether the predictive effect of achievement goal orientation on learning anxiety is realized through peer interaction. Decades of research indicate that peer interaction, where individuals discuss or work on a task collaboratively, may be beneficial for children’s and adolescents’ learning (Tenenbaum et al., 2019), and it has been found that the relationship between peer interaction and learning remained stable over time even though individual student behavior did not (Webb, 1989).

To investigate the above question, this study aimed to explore the effects of achievement goal orientation and peer interaction on learning anxiety as well as the mediating effect of peer interaction on the relationship between achievement goal orientation and learning anxiety. We hypothesized that the predictive effect of achievement goal orientation on adolescent learning anxiety is realized through the mediating factor of peer interaction.

## 2. Materials and methods

### 2.1. Procedure

The Achievement Goal Approach Scale, Learning Anxiety Scale, and Peer Interaction Scale were administered to participants in classes of 40 to 50 students. For each class, a trained psychology researcher administered the survey questionnaire. The procedure took approximately 10 min. Questionnaires were collected immediately once completed.

### 2.2. Participants

Five hundred and twenty-four students from three public junior high and senior high schools in China voluntarily participated in the questionnaire survey. Informed consent was obtained from parents, where parents received an information letter and were given the opportunity to exempt their child from participating, and the privacy rights of human subjects were observed throughout the project. Participants received no reward for their participation. The study was conducted in accordance with the code of ethics and had passed all relevant ethical review processes of the Academy of Education, Shandong Women’s University. After reduction and screening, 470 valid questionnaires (89.69%) were included in the analysis. The participants included 213 boys (45.32%) and 257 girls (54.68%) and 191 junior high school students (40.64%, 83 boys) and 279 senior high school students (59.35%, 128 boys). In total, there were 61 students (13%) in junior one, 66 (14%) in junior two, and 64 (13.6%) in junior three. There were 109 students (23.2%) in senior one, 111 (23.6%) in senior two, and 59 (12.6%) in senior three. The age of participants ranged from 12 to 19 years old (*M* = 16.37, *SD* = 1.40).

### 2.3. Materials

#### 2.3.1. Achievement goal approach scale

The Chinese version of the Achievement Goal Approach Scale (Liu et al., 2006) was used to measure achievement goal approach. The 29-item scale comprises four dimensions: mastery-approach, mastery-avoidance, performance-approach, and performance-avoidance. The questionnaire begins with the sentence “I like learning because it can increase my knowledge.” Participants rate to what extent the description in the questionnaire is consistent with their actual situation using a 5-point Likert-type scale ranging from 1 (strongly disagree) to 5 (strongly agree). The results of confirmatory factor analysis indicated the scale has good validity (χ2/df = 1.94, RMSEA = 0.05, GFI = 0.91, IFI = 0.90, TLI = 0.89, and CFI = 0.90). The Cronbach’s alpha (α) of the total scale was 0.87, and the four subscales were 0.84, 0.77, 0.82, and 0.70 for mastery-approach, mastery-avoidance, performance-approach, and performance-avoidance, respectively. The split-half reliability was 0.88. McDonald’s omega values of the scale and subscales were 0.80, 0.69, 0.65, 0.80, and 0.78. The composite reliability (CR) of the scale and subscales were 0.87, 0.83, 0.78, 0.85, and 0.85. The average variance extracted (AVE) of the scale and subscales were 0.40, 0.36, 0.42, 0.41, and 0.49. An acceptable AVE value is higher than 0.5, but we can accept 0.4. Fornell and Larcker (1981) stated that if the AVE is less than 0.5 but the composite reliability is higher than 0.6, the convergent validity of the construct is still adequate.

#### 2.3.2. Learning anxiety scale

The Chinese version of the Learning Anxiety Scale is from the learning anxiety section of the Mental Health Test (Zheng et al., 2004) and has six items. The questionnaire begins with the item “I always feel nervous when my academic record is not good.” Participants respond using a 5-point Likert-type scale (1 for strongly disagree and 5 for strongly agree). The results of confirmatory factor analysis indicated the scale has good validity (χ^2^/df = 3.16, RMSEA = 0.07, GFI = 0.99, IFI = 0.99, TLI = 0.97, and CFI = 0.99). The factor loading of items ranged from 0.57 to 0.84. The Cronbach’s α, McDonald’s omega, CR, and AVE of the scale were 0.85, 0.85, 0.89, and 0.57, respectively.

#### 2.3.3. Peer interaction scale

The Chinese version of the Peer Interaction Scale, developed by Shi and Chen and revised by Wei and Jiang (2007), was used to measure peer interaction. The 18-item scale is composed of three dimensions: social anxiety, intimate relationship, and common activity. The questionnaire begins with the item “When I have difficulties, my friend will help me.” Social anxiety concentrates on the emotional reaction and avoidance behavior associated with strong anxiety, nervousness, or fear related to one or more interpersonal situations (e.g., “I feel nervous when I talk to my classmates,” “I dare not speak in front of the whole class,” and “I don’t know how to ask my classmates for help in case of difficulties”). Intimate relationship implies an emotional connection based on trust and the intimacy experienced by the individual (e.g., “My friend will help me when I am in trouble” and “My friend will share his feelings and thoughts with me”). Common activity is about completing learning or life activities with peers (e.g., “I like to go out with friends” and “I try to avoid gatherings among classmates”). Participants respond to items using a 5-point Likert scale (1 for strongly disagree and 5 for strongly agree). The results of confirmatory factor analysis indicated the scale has good validity (χ2/df = 2.17, RMSEA = 0.05, GFI = 0.94, IFI = 0.94, TLI = 0.93, and CFI = 0.94). The Cronbach’s α of the total scale was 0.80 and 0.78, 0.73, and 0.68 for social anxiety, intimate relationship, and common activity, respectively. The McDonald’s omega of the scale and subscales were 0.86, 0.83, 0.73, and 0.71. The CR of the scale and subscales were 0.88, 0.83, 0.73, and 0.72. The AVE of the scale and subscales were 0.40, 0.36, 0.38, and 0.42.

### 2.4. Data analysis

For this study, SPSS19.0 was used to estimate the reliability and construct validity of the Achievement Goal Approach Scale, Learning Anxiety Scale, and Peer Interaction Scale. Cronbach’s alpha and McDonald’s omega were used to evaluate internal consistency reliability. CR and AVE were calculated to assess various aspects of construct validity. We conducted descriptive statistics to provide a summary of students’ achievement goals, peer interaction, and learning anxiety. To describe characteristics of achievement goals, peer interaction, and learning anxiety, we performed the analysis of variance to evaluate the impact of age, gender, and the interaction of age by gender on the three variables. We conducted a Pearson correlation analysis to assess the relationships between the three variables. Finally, structural equation modeling with gender and grade as covariates *via* AMOS 17.0 was conducted to evaluate the intermediary role of peer interaction between achievement goal orientation and learning anxiety. The bootstrap method was used to obtain confidence intervals to test the indirect effect.

## 3. Results

### 3.1. Test of common method bias

In this study, the source of data is from participants’ subjective self-report, which may lead to biases between predictor variables and criterion variables (i.e., common method biases). To evaluate the reliability of the study method, Harman’s single-factor method was used to test for common method bias. Results showed that there were 19 factors with characteristic roots greater than 1, and the variation explained by the first principal factor was 8.45%, which is less than the threshold of 40%. Therefore, there was no serious common method variance in the study.

### 3.2. General description of achievement goal approach, peer interaction, and learning anxiety

Descriptive statistics and correlation results are shown in Table 1. Results showed that after controlling for gender and age, there were significant correlations among achievement approach, peer interaction, and learning anxiety. Specifically, master-approach was negatively correlated with social anxiety (*r* = −0.13, *p* < 0.01), common activity (*r* = −0.09, *p* < 0.05), and learning anxiety (*r* = −0.09, *p* < 0.05); mastery-avoidance was positively correlated with social anxiety (*r* = 0.14, *p* < 0.01) and learning anxiety (*r* = 0.39, *p* < 0.01); performance-approach was positively correlated with intimate activity (*r* = 0.17, *p* < 0.01) and learning anxiety (*r* = 0.21, *p* < 0.01); performance-avoidance was positively correlated with social anxiety (*r* = 0.17, *p* < 0.01) and learning anxiety (*r* = 0.24, *p* < 0.01).

**TABLE 1:** Descriptive statistics and correlations for achievement approach, peer interaction, and learning anxiety.

	1	2	3	4	5	6	7	8
1. Master-approach	-							
2. Master-avoidance	0.37***	-						
3. Performance-approach	0.28***	0.44***	-					
4. Performance-avoidance	-0.03	0.23***	0.18***	-				
5. Social anxiety	-0.13**	0.14**	0.03	0.17***	-			
6. Intimate activity	0.25***	0.04	0.17***	-0.05	-0.30***	-		
7. Common activity	-0.09*	0.08	0.00	0.07	0.61***	-0.08	-	
8. Learning anxiety	-0.09*	0.39***	0.21***	0.24***	0.33***	-0.08	0.21***	–
*M*	3.07	3.52	3.31	2.94	2.42	3.43	2.76	3.12
*SD*	0.49	0.66	0.65	0.46	0.73	0.67	0.60	0.90

*p < 0.05, **p < 0.01, ***p < 0.001.

Multivariate analysis of variance with grade (middle and high school) and gender (male and female) as independent variables and achievement goals, peer interaction, and learning anxiety as dependent variables was conducted. Results showed that boys’ scores for mastery-avoidance, intimate relationship, and learning anxiety were significantly lower than girls’ scores [*F*_(1,466)_ = 7.37, *p* < 0.05; *F*_(1, 466)_ = 11.69, *p* < 0.05; *F*_(1, 466)_ = 7.81, *p* < 0.05], and boys’ scores for social anxiety and common activity were significantly higher than girls’ scores [*F*_(1, 466)_ = 4.98, *p* < 0.05; *F*_(1, 466)_ = 11.70, *p* < 0.05]. The score for performance-approach of middle school students was significantly lower than that of high school students [*F*_(1, 466)_ = 12.82, *p* < 0.001], and the scores for performance-approach and learning anxiety of middle school students were significantly higher than that of high school students [*F*_(1, 466)_ = 5.90, *p* < 0.05; *F*_(1, 466)_ = 5.90, *p* < 0.05]. There was no significant difference between gender and grade for the other variables, and the interaction between gender and grade was also not significant.

### 3.3. Direct effect of achievement approach on learning anxiety

Structural equation modeling was used to analyze the relationships between variables. The sub-dimensions of the achievement approach were the exogenous latent variables, learning anxiety was the endogenous latent variable, and gender and grade were covariates. The overall fitting indexes of the model were as follows: χ^2^/df = 0.33, RMSEA = 0.00, NFI = 0.99, GFI = 1.00, IFI = 1.00, and CFI = 1.00. The path coefficients for mastery-approach, mastery-avoidance, performance-approach, and performance-avoidance were −0.27 (*p* < 0.001), 0.42 (*p* < 0.001), 0.08 (*p* > 0.05), and 0.13 (*p* < 0.01), respectively. By comparison with nested models (Lin, 2008), if the path coefficients of each dimension of the achievement approach to learning were equal, the change compared with the original model was △χ^2^ = 80.99 (*p* < 0.001). These results indicate that the achievement approach had a significant predictive effect on learning anxiety, and the effect of each dimension was different.

### 3.4. Mediating effect of peer interaction

**FIGURE 1:**
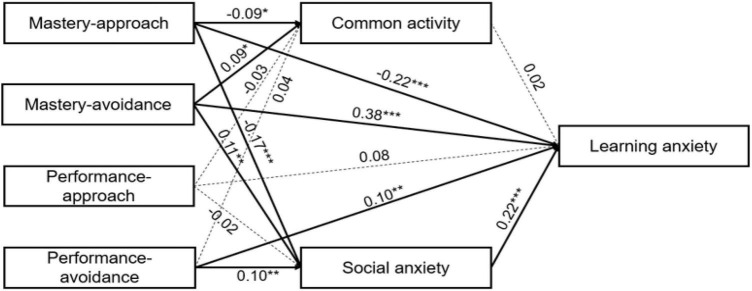
Structural equation model predicting adolescents’ learning anxiety. This structural equation model predicts adolescents’ learning anxiety from achievement orientation, with mediating effect of three dimensions of the peer interaction. To make the model clear, the control variables (gender and grade), another insignificant variable (intimate relation), and the path coefficients are not shown in figure. Statistics are standardized regression coefficients. Dotted lines represent non-significant relations; bold lines represent significant indirect paths. *p < 0.05, **p < 0.01, ***p < 0.001.

After controlling for gender and age, social anxiety, intimate relationship, and common activity were included as mediating variables in the model, and the results are shown in Figure 1. The overall fitting indexes of the model were as follows: χ^2^/df = 1.77, RMSEA = 0.04, NFI = 0.98, GFI = 0.99, IFI = 0.99, and CFI = 0.99. Compared to the direct effect, the variation in achievement approach to learning anxiety decreased but did not reach a significant level. Findings show that achievement goal orientation had a positive effect on learning anxiety (mastery-approach β = −0.22, *p* < 0.001; mastery-avoidance β = 0.38, *p* < 0.001; performance-approach β = 0.08, *p* > 0.05; performance-avoidance β = 0.10, *p* < 0.01). The four dimensions of the achievement approach had different significantly predictive effects on social anxiety (mastery-approach β = −0.17, *p* < 0.001; mastery-avoidance β = 0.11, *p* < 0.01; performance-approach β = −0.02, *p* > 0.05; performance-avoidance β = 0.10, *p* < 0.01). The four dimensions of the achievement approach had different significantly predictive effects on common activity (mastery-approach β = −0.09, *p* < 0.05; mastery-avoidance β = 0.09, *p* < 0.05; performance-approach β = −0.03, *p* > 0.05; performance-avoidance β = 0.04, *p* > 0.05). The four dimensions of the achievement approach had different significantly predictive effects on intimate relationships (mastery-approach β = 0.25, *p* < 0.001; mastery-avoidance β = −0.11, *p* < 0.01; performance-approach β = 0.15, *p* < 0.01; performance-avoidance β = −0.05, *p* > 0.05). The three dimensions of peer interaction had different predictive effects on learning anxiety, and only social anxiety in peer interactions had a significant predictive effect (social anxiety β = 0.22, *p* < 0.001; common activity β = 0.02, *p* > 0.05; intimate relationship β = 0.03, *p* > 0.05). To make the model clear, path coefficients of the control variables (gender and grade) and non-significant variables (e.g., intimate relationship) are not shown in Figure 1.

Based on the research by Wen and Ye (2014), the bootstrap method was used to obtain confidence intervals to test the indirect effect (see Table 2). Because the confidence intervals of the indirect effects of the three paths do not contain zero, it indicates that social anxiety played a mediating role in the influence of mastery-approach, mastery-avoidance, and performance-avoidance on learning anxiety, accounting for 9.91, 14.53, and 9.09% of the total effect, respectively. The confidence interval of the indirect effect of common activity on learning anxiety contains zero (−0.063, 0.102), so the mediating effect was not significant.

**TABLE 2:** The mediating effect of peer interaction.

	Indirect effect	SE	95% CI	% of the total effect
M-ap→SA→LA	−0.037	0.013	[−0.067, −0.016]	9.91
M-av→SA→LA	0.024	0.010	[0.008, 0.053]	14.5
P-av→SA→LA	0.022	0.009	[0.006, 0.042]	9.09

CI, confidence interval; M-ap→SA→LA, social anxiety as mediator effect on the influence of mastery-approach goal orientation on learning anxiety; M-av→SA→LA, social anxiety as mediator effect on the influence of mastery-avoidance goal orientation on learning anxiety; P-av→SA→LA, social anxiety as mediator effect on the influence of performance-avoidance goal orientation on learning anxiety.

## 4. Discussion

The main purpose of this study was to explore the characteristics of various achievement goals among adolescent Chinese students and whether the goals related differently to peer interaction and learning anxiety. The indirect relationship between achievement goal orientation and learning anxiety *via* peer interaction were also examined. Our results on the relationship between achievement goals and learning anxiety were in line with the results of previous studies (Linnenbrink-Garcia and Barger, 2014; Xiong, 2022) and proved to be robust when controlling for students’ gender and grade, as well as the subject domain in which the constructs were assessed. Specifically, the achievement goal approach (including mastery-approach, mastery-avoidance, and performance-avoidance) significantly predicted adolescents’ learning anxiety, and these relationships were mediated by social anxiety (but not common activity or intimate relationship) in peer interactions.

### 4.1. Characteristics of achievement goal approach, peer interaction, and learning anxiety

Gender is one of the most widely researched variables in achievement studies, but only a few studies have examined gender differences in achievement goal orientation (Meece et al., 2009), and studies that have focused on gender differences are somewhat inconsistent in their findings (Middleton and Midgley, 1997; Duchesne et al., 2014; Bahar et al., 2018; Guan et al., 2023). In this study, we found significant gender differences for mastery-avoidance goal orientation, peer interaction, and learning anxiety (i.e., girls had slightly stronger mastery-avoidance goals, learning anxiety, and intimate relationships in peer interactions than their male counterparts, and lower social anxiety and common activity in peer interactions). Achievement goal results were partially consistent with some studies (Cecchini-Estrada et al., 2011; Lochbaum et al., 2019) and partially inconsistent with other previous findings (Meece et al., 2006b; Bahar et al., 2018; Guan et al., 2023; Hemi et al., 2023). Gender differences in learning anxiety were consistent with some previous research (Hill and Sarason, 1975; Maccoby and Jacklin, 1998; Ho et al., 2000), whereas other studies indicated there were no or fewer gender differences (Elsequest et al., 2010; Olmez and Ozel, 2012; Goetz et al., 2013). Nevertheless, a range of previous studies explored the relationship between gender and learning anxiety and tended to provide evidence that girls suffered more from anxiety (e.g., specifically math anxiety) in the academic context (Yüksel-Sahin, 2008; Devine et al., 2012).

Some studies report significant gender differences favoring female students except for performance goal orientation (Meece et al., 2006b; Hemi et al., 2023), while other studies report non-significant findings (Greene et al., 2002; Sahin et al., 2016; Guan et al., 2023). A possible explanation is that mastery-avoidance goals require gaining some level of expertise or skills that one would wish to maintain, and unlike male students, female students’ motivation to succeed may be more directed toward developing competence than toward intentions to outperform others or hiding their incompetence (Duchesne et al., 2014). As such, female students may perceive more positive learning when they are disadvantaged in high school classrooms. Based on the above, it is reasonable to assume that there would be gender differences in the relationship between mastery-avoidance goal orientation and learning anxiety in students. To date, inconsistent results can be attributed to different participant samples or forms of achievement; for example, previous studies focused singularly on students in middle school, high school, college, or physical activity settings. The present findings showed there were gender differences in how students’ goals related to various learning-related variables.

The finding that gender differences were significant for social anxiety and common activity is consistent with a previous study (Tenenbaum et al., 2019). Thus, the current results that differ from previous studies may be the different forms of knowledge. Gender is a factor that might influence the effectiveness of peer interactions (Strough et al., 2001; Leman and Tenenbaum, 2017). It is possible that the present forms of knowledge (e.g., conceptual vs. procedural scientific knowledge) may suit boys’ interactions (e.g., common activity) better than girls’ interactions (Leman et al., 2016). Yu et al. (2021) found half of the boys were doing fine in school, while half of the girls displayed worrying patterns of motivation, engagement, and achievement. Furthermore, a recent study revealed that girls had an increased tendency to give up and self-handicap after the transition to secondary school and might experience more positive relationships with their teachers and peers to protect them against the decline in motivation (Burns et al., 2019). In contrast, male students who feel pressured to appear emotionally detached and self-reliant were found to adopt a surface approach to learning (Marrs, 2016) and avoid seeking help in the classroom (Leaper et al., 2019). For the above reasons, the social anxiety and common activities in male students would be greater than in female students, but the learning anxiety and intimate relationships in peer interactions would be less than that of female students.

Another widely examined variable associated with achievement goal orientation, learning anxiety, and peer interaction is student grade level. Like gender, there have been mixed findings with respect to grade-related differences in the four achievement goal orientations. This study showed grade differences in performance-avoidance goal orientation (e.g., performance-approach of junior high school students was significantly lower than that of senior high school students, but performance-avoidance and learning anxiety of junior high school students were significantly higher than that of senior high school students), which is partially consonant with some studies (Meece et al., 2006a; Guan et al., 2023) and inconsonant with others (Cecchini-Estrada et al., 2011; Guan et al., 2020). Guan et al. (2023) revealed that age, as a continuous variable, had a significant and positive effect on students’ mastery-approach goals, performance-approach goals, and mastery-avoidance goals, and a negative effect on students’ performance-avoidance goals. However, we only found performance-avoidance goals were negatively related to students’ age and performance-approach goals were positively related to students’ age. We attributed this result to the following possible reasons. First, as with gender, all previous grade-related achievement goal studies using the 2 × 2 model only focused on grade levels within the same school level and physical activity setting (Guan et al., 2023), while our study investigated a wider array of grade levels across achievement settings at multiple school levels (e.g., junior high school vs. senior high school). Second, the participants were Chinese students, and senior high school students had more pressure to attend college *via* the college entrance examination than junior high school students. Therefore, senior high school students may not pay attention to the accumulation of experience rather their focus may be the pursuit of good test results, focusing on winning or demonstrating superior ability relative to others. Consequently, they would be more likely to have stronger performance-approach goals than junior high school students. Simultaneously, junior high school students were more likely to endorse performance-avoidance goals; they primarily focused on escaping failure by concealing their relative incompetence, especially when compared with or in front of peers. Consequently, they would have more learning anxiety. Moreover, this result confirmed the correlation between performance-avoidance goal orientation and learning anxiety. This positive link was stronger in the studies within a senior high school group (Zhang et al., 2019).

The research on gender differences in achievement goal orientation, peer interaction, and learning anxiety has focused on understanding mastery-avoidance goals, social anxiety, and learning anxiety. Taken together, research on gender differences in achievement goal orientation and learning anxiety is inconclusive and equivocal. Patterns of gender differences depend on the methodology used, academic domain, type of achievement task, and research setting. Additionally, when gender differences are found, they tend to be small in magnitude and not a strong predictor of behavioral responses (Eccles et al., 1983; Parsons et al., 1984).

### 4.2. Achievement goal orientation predicts learning anxiety in adolescents

This study found that achievement goal orientation had a significant predictive effect on learning anxiety, which is consistent with previous studies (Dull et al., 2015; Niemivirta et al., 2019; Liu et al., 2020; Miller et al., 2021; Xiong, 2022). Achievement goal orientation theory explains why students strive to perform well academically from the perspective of learning motivation (Ames, 1992). According to this theory, adolescents’ achievement goal orientation structure will affect their personal goal orientation choice, and also affect their educational results. Each achievement goal orientation produces unique educational results.

First, master-avoidance and performance-avoidance goal orientations positively predicted learning anxiety, which is consistent with previous studies (Elliot and McGregor, 2001; Putwain and Symes, 2012; Daumiller and Dresel, 2020; Zafarani et al., 2022) and confirms the hypothesis of Linnenbrink and Pintrich (2000). Linnenbrink and Pinrich proposed that mastery-approach goal is connected with low anxiety, mastery-avoidance goal and performance-approach goal are connected with medium anxiety, and performance-avoidance goal is connected with high anxiety (Pekrun et al., 2009; Putwain and Symes, 2012; Skaalvik, 2018). Students with an avoidance goal orientation tend to focus on the possibility of failure and ignore the positive side of the event (Elliot and Pekrun, 2007). Moreover, from the absolute value of the correlation coefficient, the correlation between mastery-avoidance goal and learning anxiety is greater than that between performance-avoidance goal and learning anxiety, which indicates that for middle school students, mastery-avoidance goal is connected with higher learning anxiety. This is consistent with some research results (Liu and Guo, 2003; Liu et al., 2020).

Second, we found mastery-approach could negatively predict learning anxiety, which is also consistent with previous studies (Elliot and McGregor, 1999; Shih, 2005; Goetz et al., 2016; Palazzolo, 2020; Sun et al., 2020). However, the effect of performance-approach goal orientation on the prediction of learning anxiety was not significant although they were positively correlated, which has been found in some studies (Smith et al., 2002; Mattern, 2005; Hong et al., 2018; Liu et al., 2020) but differed from the results of other studies (Cooper, 2014; Dull et al., 2015; Tu and Yang, 2021). It may be that students with a high performance-approach goal orientation are more concerned about their performance than their peers, so they have more worries about learning. However, such students tend to perceive a high level of teachers’ emotional support (Lerang et al., 2019), and the focus on success might replace the fear of failure (Putwain and Symes, 2012). Based on the above reasons, this study did not find performance-approach goal orientation to have a significant predictive effect on learning anxiety. Results for the performance-approach orientation were more mixed (Tuominen-Soini et al., 2012; Miller et al., 2021). It may be that students with performance-approach goal orientation were self-efficient and so confident in their academic performance that it was difficult to detect anxiety through the questionnaire (Shih, 2005).

### 4.3. Mediating effect of peer interaction

This study found that peer interaction played a mediating role in the influence of achievement goal orientation on learning anxiety, which partially confirmed previous studies (Tenenbaum et al., 2019; Liu et al., 2020). Students who employed a mastery-approach orientation were more likely to partake in peer interaction, which is in accordance with previous empirical findings (Elliot et al., 2016; Miller et al., 2021). DuBois et al. (1992), Harter (1996), Senko (2019) and found correlations between students’ achievement goals and peer interaction. Relevant to the present study, achievement goal has been found to influence social interactions with peers on academic tasks (Darnon et al., 2012). Achievement goal popularity norms played a role in friendship processes; conversely, friendship influence on achievement took place in classrooms with high mastery goal popularity norms (Laninga-Wijnen et al., 2018). Positive peer interaction could improve achievement motivation and involvement in class activities (Deci, 1992). Piaget (e.g., Piaget, 1932) viewed peer interaction, distinct from adult-child interaction, as an important means of promoting intersubjectivity and subsequent social development. Adolescence is a period of development and transition. In this period, as the focus of individual interpersonal communication gradually shifts from parents to peers, peer communication, as social support, has a strong impact on individual personality development. Teenagers’ negative peer interaction may lead to school maladjustment, high-risk behaviors, and behavioral disorders (Podsakoff et al., 2012). Simultaneously, adolescents start to reduce their attachment to adults and rely on peer interaction to build and maintain a positive self-concept. Adolescents can share their thoughts and feelings with each other, for example, properly revealing themselves and establishing close relationships to better adapt to society. In both, the notion of intersubjectivity, that is, shared meaning between interactional partners is key in explaining possible learning benefits from peer interaction (Tenenbaum et al., 2019). In particular, in this approach we explored the mechanisms underpinning learning through peer interaction.

From a Piagetian view, peer interaction facilitates learning because intersubjectivity creates socio-cognitive conflict. Then to restore cognitive balance, adolescents would expect greater learning where the task specifically requires them to reach a consensus through interaction. Alternatively, self-determination theory (Deci and Ryan, 1985) proposes that individuals have inherent basic psychological needs, including autonomy, competence, and relatedness, for the purpose of self-motivation and personality integration (Deci and Ryan, 2008; Mageau et al., 2015). Deci and Ryan (2000) showed that when a person’s (relatedness) need is fulfilled, it will further promote the other two needs (autonomy and competence) to be fulfilled. Therefore, peer interaction will promote the satisfaction of an individual’s basic psychological needs, which can promote their positive development, enable them to experience positive emotions, and improve their self-evaluation and life satisfaction. However, anxiety is easy to develop if peers are in conflict, or they are in a tense relationship (Berndt, 2002). Kadir (2018) found that poor peer interaction among middle school students affected their learning anxiety, and it is widely believed that adolescents who are in negative peer relationships are at high risk of academic problems (Goodenow, 1993). Therefore, the mediating effect of peer interaction on the relationship between achievement goal orientation and learning anxiety should not be ignored (Simões and Alarcão, 2014). If there is a high conflict with a peer or both parties have strong dominance, anxiety can easily occurre (Berndt, 2002). Wang and Ding (2003) found that interpersonal communication among middle school students further alleviated the problem of anxiety. Moreover, the general view is that adolescents who lack good relationships with adults and peers are a high-risk group for academic problems (Goodenow, 1993). Therefore, the mediating effect of peer communication on achievement goal orientation and learning anxiety cannot be ignored.

Students with a mastery-approach goal tend to have high autonomy and pay attention to the acquisition of new knowledge and skills (Alhadabi and Karpinski, 2020). They pursue happiness by learning and usually show a high level of self-efficacy and a low level of learning anxiety. However, when students face adverse events or negative peer interactions, their autonomy can be decreased by social anxiety (Deci and Ryan, 2000) and can thereby indirectly cause learning anxiety. For students with a mastery-avoidance goal orientation, their tendency to avoid incompetence is greater than the desire to achieve success, and they give more attention to avoiding the negative effects of failure (Elliot and McGregor, 2001). Therefore, peer interactions might increase their autonomy to avoid failure, and thereby reduce anxiety. It has been confirmed that communication needs as human instinctive psychological needs (Deci and Ryan, 2000) and emotional links between individuals and others affect people’s learning fields. Even in an individual’s life, safe interpersonal relationships can provide a good environment for the development of internal motivation. When relationship needs are not met, social anxiety will occur, which will have an important impact on learning across different areas of the individual’s life and trigger the generation of learning anxiety. Therefore, social anxiety plays a mediating role in mastering and avoiding goal orientations and in influencing learning anxiety. Students with a performance-avoidance goal orientation pay more attention to avoiding clumsy and incompetent performance, usually showing a low level of self-efficacy. They are afraid of showing insufficient ability and try to avoid damaging their future and sense of value; thus, they usually feel anxious and nervous about tests and evaluations. Furthermore, students with a performance-avoidance goal orientation value friends’ evaluations to build their identity (Hergovich et al., 2002); thus, pressure in peer interactions might also indirectly affect their anxiety in learning. In the analysis of the mediating effect of peer interaction, we found that social anxiety accounted for the highest proportion of the mediating effect on the impact of performance-avoidance goals on learning anxiety. The results suggest that compared with other goal orientations, social anxiety has a greater impact on performance-avoidance goal orientation because the sense of tension and powerlessness induced by performance-avoidance can impede learning.

### 4.4. Educational implications of the study

The findings support previous studies but also provide new insights into the relationship between achievement goal orientation and learning anxiety. The major contribution of this study to the field of learning anxiety is that it examines the use of peer interaction in reducing learning anxiety and investigates the relationship between achievement goal orientation and learning anxiety. Specifically, this study discusses the influence of the four types of achievement goal orientation on learning anxiety and describes the mediating effect of peer interaction. Based on previous empirical research, critical predictors of peer interaction include the level of social anxiety and common activity. Hypotheses about important but neglected aspects of peer interaction that may predict learning anxiety are discussed. Because adhering to social distancing may be particularly challenging for adolescents for whom interaction with peers is especially important, we argue that young people’s capacity to encourage each other to reduce learning anxiety should be harnessed. In addition, mediation analyses indicate that peer interaction is more effective when adolescents are not worried or nervous about communicating than when they have intimate relationships with peers.

This result reminds us that it is important to strengthen the training of students to establish a mastery-approach goal orientation and guide students to pursue the improvement of competence in learning. Meanwhile, teachers and parents need to pay attention to students’ peer interactions and create conditions conducive to peer interactions (Neri Tejada et al., 2022). Students may need guidance on how to deal with communication difficulties to relieve communication anxiety and alleviate peer anxiety. This will allow adolescents to satisfy societal needs and contribute to their mental health development. In general, our results offer evidence for the importance of peer interaction when examining student learning anxiety and the relationship between achievement goal orientation and learning anxiety, but we stress that peer interaction is a critical predictor. The findings extend theoretical considerations by teasing out the process of peer interaction affecting the relationship between achievement goal orientation and learning anxiety. Additionally, the results have practical implications for the effective use of peer interaction to reduce learning anxiety.

### 4.5. Limitations of the study

Despite the novel findings and implications of this study, several limitations should be clarified. First, this study adopted a cross-sectional design, which could not explore the development of achievement goal orientation and peer interaction on learning anxiety. Second, the present study was conducted using students’ self-report. If we used other additional evaluation methods (e.g., a parent’s report or teacher’s report), our findings would be enriched. Furthermore, this study did not measure gender and grade differences in the link between achievement goal orientation, peer interaction, and learning anxiety and only reported the link based on a mixed-gender sample. To develop a better understanding of this link, additional details (e.g., multiple-group analysis) could be given for a range of relationships.

Future studies could extend our findings by exploring whether the implications based on our results can be applied in an educational context. Additionally, follow-up research or an experimental design can be used to further determine the causal relationship and internal mechanism between variables. As stated above, the combination of multiple evaluation methods such as parent’s report or teacher’s report should be considered. Lastly, to develop a better understanding of the effect of gender and grade on the link between achievement goal orientation, peer interaction, and learning anxiety, additional research should be conducted; these explorations would help researchers and educators to adopt eligible methods to alleviate learning anxiety and promote the learning performance of students.

## 5. Conclusion

There were significant gender differences in mastery-avoidance goal orientation, peer interaction, and learning anxiety, and there were grade differences in performance-approach and performance-avoidance goal orientations. Mastery-approach, mastery-avoidance, and performance-avoidance goal orientations directly predicted learning anxiety. Social anxiety in peer interactions had a mediating effect on the influence of mastery-approach, mastery-avoidance, and performance-avoidance goal orientations on learning anxiety.

## Data availability statement

The raw data supporting the conclusions of this article will be made available by the authors, without undue reservation.

## Ethics statement

This study was approved by the Ethical Review Board of the Academy of Education, Shandong Women’s University. Written informed consent to participate in this study was provided by the participants’ legal guardian/next of kin. Written informed consent was obtained from the individual(s), and minor(s)’ legal guardian/next of kin, for the publication of any potentially identifiable images or data included in this article.

## Author contributions

HK generated ideas for the study, contributed to the study design, and performed the formal analysis. GW conducted the experiments and collected data. HK and TL drafted the manuscript. TL, HK, and DC provided edits and revisions. All authors approved the final version of the manuscript for submission.
